# Variation in Use of Surgical Care During the COVID-19 Pandemic by Surgical Urgency and Race and Ethnicity

**DOI:** 10.1001/jamahealthforum.2021.4214

**Published:** 2021-12-23

**Authors:** Thomas C. Tsai, Ava Ferguson Bryan, Ning Rosenthal, Jie Zheng, E. John Orav, Austin B. Frakt, Jose F. Figueroa

**Affiliations:** 1 Department of Surgery, Brigham and Women’s Hospital, Boston, Massachusetts; 2 Center for Surgery and Public Health, Boston, Massachusetts; 3 Department of Health Policy and Management, Harvard T.H. Chan School of Public Health, Boston, Massachusetts; 4 Department of Surgery, The University of Chicago, Chicago, Illinois; 5 Premier Applied Sciences, Premier, Inc, Charlotte, North Carolina; 6 Division of General Internal Medicine and Primary Care, Brigham and Women’s Hospital, Boston, Massachusetts; 7 Boston VA Healthcare System, Boston, Massachusetts; 8 Boston University School of Public Health, Boston, Massachusetts

## Abstract

**Importance:**

The extent of the disruption to surgical care during the COVID-19 pandemic has not been empirically characterized on a national level.

**Objective:**

To characterize the use of surgical care across cohorts of surgical urgency during the COVID-19 pandemic, and to assess for racial and ethnic disparities.

**Design, Setting, and Participants:**

This was a retrospective observational study using the geographically diverse, all payer data from 767 hospitals in the Premier Healthcare Database. Procedures were categorized into 4 cohorts of surgical urgency (elective, nonelective, emergency, and trauma). A generalized linear regression model with hospital-fixed effects assessed the relative monthly within-hospital reduction in surgical encounters in 2020 compared with 2019.

**Main Outcomes and Measures:**

Outcomes were the monthly relative reduction in overall surgical encounters and across surgical urgency cohorts and race and ethnicity.

**Results:**

The sample included 13 175 087 inpatient and outpatient surgical encounters. There was a 12.6% relative reduction in surgical use in 2020 compared to 2019. Across all surgical cohorts, the most prominent decreases in encounters occurred during Spring 2020 . For example, elective encounters began falling in March, reached a trough in April, and subsequently recovered but never to prepandemic levels (March: −26.8%; 95% CI, −29.6% to −23.9%; April: −74.6%; 95% CI, −75.5% to −73.5%; December: −13.3%; 95% CI, −16.6%, −9.8%). Across all operative surgical urgency cohorts, White patients had the largest relative reduction in encounters.

**Conclusions and Relevance:**

As shown by this cohort study, the COVID-19 pandemic resulted in large disruptions to surgical care across all categories of operative urgency, especially elective procedures. Racial and ethnic minority groups experienced less of a disruption to surgical care than White patients. Further research is needed to explore whether the decreased surgical use among White patients was owing to patient discretion and to document whether demand for surgical care will rebound to baseline levels.

## Key Points

**Question:**

To what extent did the COVID-19 pandemic reduce access to surgical care, and were racial and ethnic minority groups more likely to have reduced access to surgical care?

**Findings:**

In this cohort study of more than 13 million inpatient and outpatient surgical encounters in 767 US hospitals in a hospital administrative database, surgical use was 13% lower in 2020 compared with 2019, with the greatest decrease concentrated in elective surgical procedures. While Black and Hispanic patients experienced a reduction in surgical encounters, White patients experienced the greatest reduction in surgical encounters.

**Meaning:**

Despite severe and persistent disruptions to health systems during the COVID-19 pandemic, racial and ethnic minority groups did not experience a disproportionate decrease in access to surgical care.

## Teaser

This cohort study examines the use of surgical care across cohorts of surgical urgency during the COVID-19 pandemic and assesses whether there are racial or ethnic disparities in care.

## Introduction

The coronavirus disease 2019 (COVID-19) pandemic has had a profound effect on the US health care delivery system. The COVID-19 pandemic led to unprecedented numbers of individuals seeking emergency, inpatient, and intensive care. Cancellation and postponement of elective surgical procedures emerged as a primary mechanism to generate hospital surge capacity. State-mandated restrictions of elective surgery occurred in 2 waves, during the spring and winter of 2020. To date, the scale of deferred surgical care on a national level remains unknown.

Equally importantly, there is concern by policy makers and clinicians that deferment of surgical care may have been uneven across racial and ethnic groups, reflecting potential unconscious bias in discretionary scheduling of elective procedures. Both the postponement and the resumption of surgical care during the COVID-19 pandemic relied on surgical triage algorithms that prioritized nonelective over elective surgical operations.^1,2,3,4^ It is unknown whether these surgical triage decisions may have had the unintended consequence of reducing access to care for racial and ethnic minority groups. Given the disproportionate burden of COVID-19 on racial and ethnic minority groups and historical racial and ethnic disparities stemming from systemic racism and unequal treatment, equitable access to necessary care is a national priority.

Using recent and nationally representative data, we sought to answer 3 questions to provide empirical insight on the cancellation and resumption of surgical care in the US during the COVID-19 pandemic prior to the wide availability of vaccinations. First, what was the degree of decrease in surgical encounters during the COVID-19 pandemic and did surgical care recover to pre-pandemic levels? Second, did the relative reduction of surgical service use vary across elective, nonelective, emergency, and trauma surgical cohorts? Last, did the relative reduction of surgical use vary by racial and ethnic groups?

## Methods

### Patient Data

We analyzed the Premier Healthcare Database (PHD), an all-payer, geographically diverse deidentified hospital administrative database with more than 1 billion patient encounters representing approximately 25% of inpatient discharges in the United States.^5^ The hospitals included in the PHD are a subset of hospitals that are on the Premier Quality Advisor Platform and have agreed to make their data available for research. Compared with hospitals included in the American Hospital Association (AHA) Annual Survey, the PHD has a greater proportion of hospitals located in the South (43.9% vs 37.4%), rural hospitals (29.8% vs 24.1%), nonteaching hospitals (71.7% vs 59.2%), and hospitals greater than 400 beds (19.6% vs 10.4%) (eTable 1 in the Supplement). The PHD contains patient-level and visit-level data from hospital discharge files where a patient can be followed within a facility using a unique identifier. The PHD contains information on patient demographics; diagnoses; billed laboratory and diagnostic services; and billed medications and procedures. This data set has been used to rapidly identify the burden of COVID-19 and risk-factors associated with COVID-19 inpatient mortality by many studies.^6,7,8^ We identified 767 hospitals with continuous monthly data submissions from January 1, 2019, to December 31, 2020.

This study was approved by the Institutional Review Board of the Harvard T.H. Chan School of Public Health. Because the PHD contains deidentified data, informed consent of study participants was not pursued. This study followed the Strengthening the Reporting of Observational Studies in Epidemiology (STROBE) reporting guideline.

### Variables

The primary outcome of this study was the within-hospital relative reduction of total surgical encounters (across inpatient and outpatient centers) in 2020 compared with the baseline year of 2019. Four types of surgical urgency cohorts were assessed: elective, nonelective, emergency, and trauma.

A subset of operations representing the elective, nonelective, and emergency surgical cohorts were identified using the *International Statistical Classification of Diseases, Tenth Revision, Clinical Modification (ICD-10-CM) *procedure codes, Current Procedural Terminology (CPT), and *ICD-10-CM* diagnosis codes: elective (bariatric surgery, joint replacement, hernia repair, breast reconstruction, myomectomy/hysterectomy, and ostomy closure), nonelective (mastectomy, prostatectomy, pulmonary lobectomy, colectomy for cancer, aortic valve replacement), and emergency (appendectomy, breast incision and drainage, cholecystectomy, colectomy for diverticulitis or infectious colitis, incarcerated hernia repair, bowel resection for ischemia or obstruction) (eTable 2 in the Supplement). We chose these procedures because they represent a spectrum of surgical specialties with varying clinical priorities performed across academic and community hospitals; additionally, the emergency surgical cohort represents a validated set of procedures representing a high burden of operative acute surgical illness.^9^ Nonelective surgical encounters consisted primarily of cancer and cardiovascular operations based on surgical priority criteria at many facilities during the COVID-19 pandemic.^1,2^

The trauma cohort was defined by *ICD-10-CM* diagnosis codes (eTable 2 in the Supplement). Encounters for trauma included all operative and nonoperative trauma encounters, including hospital admissions and emergency department evaluations. We included nonoperative trauma encounters, as 88.8% of trauma encounters in the US are nonoperative.^10^ The trauma cohort served as a negative control to assess nondiscretionary health care use by patients. Changes in the volume of trauma encounters could further reflect potential constraints on inpatient surgical censuses, as trauma encounters are typically managed by general, acute care, and trauma surgeons who were often clinically deployed to provide medical or intensive care during the pandemic.^11,12^

Race and ethnicity were categorized as Hispanic, non-Hispanic Black, and non-Hispanic White patients.^13^ Patients of Asian, other, or unknown race or ethnicity were classified by the authors as other. Additional patient-level variables included age, gender, and insurance status. Hospital-level variables included size, teaching status, region, and urban-rural status.

### Statistical Analyses

The main unit of analysis was the hospital-month. For all analyses, we employed a hospital fixed effects approach, which allows each hospital to serve as its own control and adjusts for all time invariant hospital-level covariates.^14^ Given potential case-mix variation across hospitals, the hospital fixed effects approach allows within-hospital comparisons of changes in overall surgical encounters and changes in surgical encounters by race and ethnicity.

We first developed a generalized linear model assuming a gamma distribution and a log-link, to assess the relative reduction of encounters by hospital, by month, for calendar year 2020 vs 2019. The gamma model was chosen to accommodate the skewed distribution of admissions, and the log-link allowed estimation of the relative reduction in admissions. We then calculated the relative reduction in surgical encounters as a proportion from the comparable monthly baseline in 2020 vs 2019. We chose the relative reduction as our main outcome to generalize our findings across varying hospital sizes and admissions. Given that the waves of COVID-19 pandemic varied across regions of the US, we repeated our model estimates but stratified by the US Census regions of Northeast, Midwest, South, and West.

We repeated this analytic approach for our secondary outcomes of elective, nonelective, emergency, and trauma surgical encounters. We then repeated this model but stratified by inpatient and outpatient surgical encounters. Finally, we repeated the same analytic approach to assess for changes in surgical encounters by race and ethnicity, stratified by surgical cohorts. All analyses were conducted in SAS (version 9.4) with 2-tailed *t* tests, where applicable, and a *P* value of .05 to establish statistical significance.

### Sensitivity Analyses

We performed a sensitivity analysis to assess the robustness of our results. We repeated the analyses without hospital fixed effects and controlling for hospital structural characteristics, COVID-19 burden, and region. Given the variation in waves of COVID-19 across various regions of the US, this sensitivity analysis included fixed effects at the region level.

## Results

### Patient and Hospital Characteristics

There were 13 175 087 surgical encounters identified in our sample across 2019 and 2020. Patient characteristics between 2020 and 2019 were largely similar (Table 1). The average proportion of patients 65 years and older was 41.1% for elective surgeries, 58.0% for urgent surgeries, 26.2% for emergency surgeries, and 22.7% for trauma encounters. The average proportion of Black patients was 10.9% for elective surgeries, 9.9% for urgent surgeries, 8.8% for emergency surgeries, and 15.0% for trauma surgical encounters (results not shown). Of the hospitals included in the analysis, 26.9% were teaching hospitals, 17.5% were large with more than 400 beds, and 68.4% were in urban areas. These hospitals were predominantly located in the South (42.2%); 29.6% were in the Midwest, 15.8% were in the West, and 12.4% were in the Northeast (Table 2).

**Table 1.** Patient Characteristics in 2020 vs 2019

Characteristic	Patient, %		*P* value^a^
	2019	2020	
Surgical urgency cohorts per hospital, No.			
Elective	756	616	<.001
Urgent	154	140	<.001
Emergency	293	249	<.001
Trauma	8396	6635	<.001
Patient characteristics			
No.	7 338 765	5 837 042	NA
Age, y			
≤18	19.6	17.4	<.001
19-49	35.9	37.0	<.001
50-64	19.2	19.7	<.001
≥65	24.7	25.4	<.001
Sex			
Male	51.8	51.3	.02
Female	47.5	48.0	.008
Unknown	0.2	0.3	.31
Race			
Black	12.9	12.8	.15
Hispanic	10.8	11.0	.55
White	69.2	68.8	.38
Other	8.6	8.7	.63
Insurance			
Medicare	26.7	27.1	.001
Medicaid	23.0	23.2	.42
Commercial	31.0	31.2	.28
Self-pay	8.9	8.4	<.001
Other^b^	9.9	9.6	.07

^a^ *P* value obtained from Pearson χ^2^ test of independence except where indicated.

^b^ In our analyses, the “other” racial and ethnic category consisted of individuals classified as “Asian,” “other,” or “unable to determine.”

**Table 2.** Hospital Characteristics

Characteristic	%
No.	767
Size, beds	
≤99	33.1
100-399	49.4
≥400	17.5
Teaching hospital status	
Nonteaching	73.1
Teaching	26.9
Region	
Northeast	12.4
Midwest	42.2
South	29.6
West	15.8
Geography	
Rural	31.6
Urban	68.4

### Trends in Overall Encounters, 2020 vs 2019

**Figure 1.**
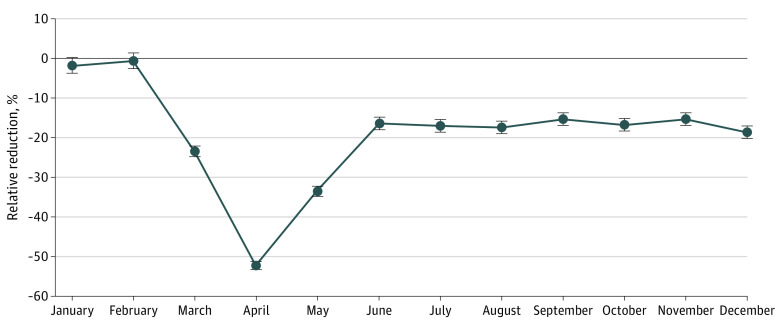
Overall Change in Surgical Encounters, 2020 vs 2019 Results from a generalized linear regression model with a log link to assess for relative reduction in hospital monthly surgical use in 2020 vs 2019.

The total number of annual surgical encounters in 2020 was 87.4% of the surgical encounters in 2019, representing a reduction of 12.6% (eFigure 1 in the Supplement). After assessing the relative reduction of overall surgical encounters by month in 2020 compared with 2019, we found a sustained reduction of surgical encounters from March to December 2020 compared with 2019. Decreased surgical encounters began in March (−23.5%; 95% CI, −24.9% to −22.1%; *P* < .001), were most pronounced in April (−52.2%; 95% CI, −53.1% to −51.3%; *P* < .001), and began to recover in May (−33.5%; 95% CI, −34.7% to −32.3%; *P* < .001), but failed to reach 2019 levels by December (−18.7%; 95% CI, −20.1% to −17.2%; *P* < .001) (Figure 1).

There were significant variations in the relative reduction of surgical encounters by region (eFigure 2 in the Supplement). Across all regions, the decrease in surgical encounters was most pronounced in April 2020. During April 2020, the Northeast had the largest overall relative reduction of surgical encounters: −66.7% (95% CI, −73.4% to −58.2%; *P* < .001).

### Relative Reduction in Trends in Surgical Encounters by Surgical Urgency, 2020 vs 2019

**Figure 2.**
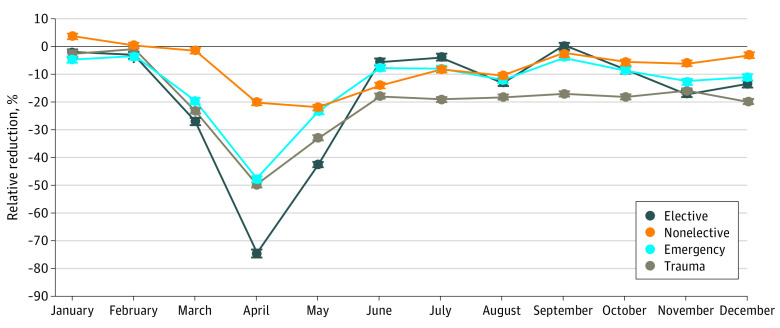
Trends in Surgical Encounters by Surgical Urgency Cohort, 2020 vs 2019 Results from a generalized linear regression model with a log link to assess for relative reduction in hospital monthly surgical use in 2020 vs 2019, stratified by surgical urgency cohort.

All surgical urgency cohorts exhibited decreased surgical encounters from March until December, but the extent of the disruption varied across surgical cohorts (Figure 2). In the spring of 2020, across all surgical cohorts, elective surgical encounters had the largest relative reduction (April: −74.6%; 95% CI, −75.5% to −73.5%; May: −42.6%; 95% CI, −44.8% to −40.3%). From June until December, the reduction in elective surgeries became less pronounced but remained sustainably below pre-pandemic levels (June: −5.5%; 95% CI, −9.1%, −1.8%; December: −13.3%; 95% CI, −16.6%, −9.8%). Both trauma and emergency surgical encounters followed similar trajectories, but with less profound variations in relative reductions across months. Nonelective surgical encounters also followed a similar trajectory but had the least profound variation in relative reductions across all months.

For inpatient surgical encounters, the most profound decrease was seen in the elective surgical cohort, with a relative reduction of −71.3% in April (95% CI, −72.5% to −70.1%; *P* < .001) and having not recovered to baseline 2019 levels within the study period (eFigure 3A in the Supplement). For outpatient surgical encounters, elective surgical procedures had a decrease in April of −67.3% (95% CI, −68.8% to −65.8%; *P* < .001). Then from June to December outpatient elective surgical encounters exceeded baseline 2019 levels by an average of 21.1% (95% CI, 15.7% to 26.8%; *P* < .001) (eFigure 3B in the Supplement).

### Relative Reduction in Surgical Encounters by Race and Surgical Urgency, 2020 vs 2019

**Figure 3.**
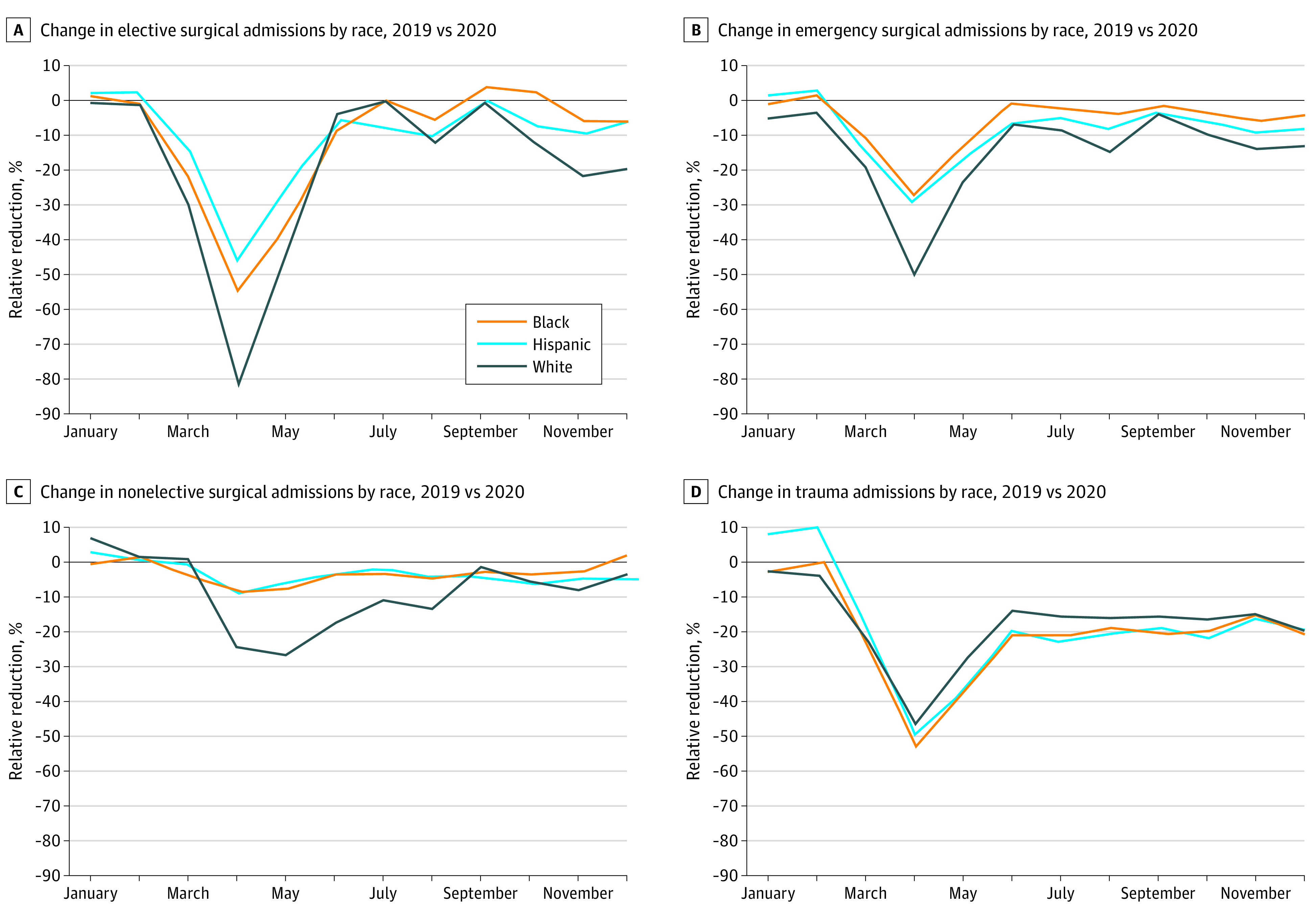
Change in Surgical Encounters by Race and Ethnicity and Surgical Urgency Cohort, 2020 vs 2019 Results from a generalized linear regression model with a log link to assess for relative reduction in hospital monthly surgical use in 2020 vs 2019, stratified by surgical urgency cohort and race and ethnicity. For ease of presentation, only results for Black, Hispanic, and White patients are displayed.

Across the 3 operative surgical urgency cohorts (elective, nonelective, emergency) and all races and ethnicities, surgical encounters decreased from spring 2019 to spring 2020. However, White patients saw the largest relative reduction in receipt of surgical care across the operative surgical urgency cohorts. For elective surgeries, the average monthly relative reduction for White patients was −18.8%, compared with −10.9% for Black patients, and −10.5% for Hispanic patients. For nonelective surgical encounters, the average monthly relative reduction for White patients was −8.4%, compared with −3.0% for Black patients, and −3.3% for Hispanic patients. Last, for emergency surgical encounters the average monthly relative reduction for White patients was −14.3%, compared with −6.1% for Black patients, and −8.7% for Hispanic patients. Across all races and ethnicities and surgical cohorts, surgical encounters saw the largest relative reduction in April, where White patients had the most prominent decrease. For ease of presentation, results are shown for Black, Hispanic, and White patients in Figure 3. Results for all racial and ethnic groups are presented in eFigure 4 in the Supplement.

### Sensitivity Analyses

In sensitivity analyses controlling for hospital characteristics, COVID-19 burden, and region, results were similar to our main findings. White patients experienced the greatest declines in surgical encounters across elective, nonelective, and emergency encounters (eTable 3, eFigure 5 in the Supplement).

## Discussion

Compared with 2019, in 2020 there was a 13% overall decrease of surgical encounters. While the relative reduction in surgical encounters was less severe in the second half of 2020, there was no rebound to the pre-pandemic baseline. Elective surgical encounters saw the greatest relative reduction of approximately 75% in April 2020. Overall, White, not racial and ethnic minority, patients were associated with the greatest decrease in surgical encounters for elective, nonelective, and emergency surgical procedures. Importantly, there were no variations in trauma encounters by race or ethnicity, suggesting equitable access to care for injury and trauma.

During the spring of 2020 there was a pronounced reduction in surgical care which became less pronounced in the second half of 2020, but never recovered to baseline 2019 levels. These findings are consistent with prior findings of reduced surgical cases during the spring and subsequently a muted recovery that still fell short of baseline use of inpatient acute care.^15,16,17^ The present study findings expand the literature documenting the decline in surgical use during COVID-19, which was previously limited to institutional case series or modeling.^18,19^ Furthermore, by stratifying across surgical urgency cohorts and including trauma encounters, the present study provides insights into discretionary and non-discretionary use of surgical care. The finding of a 50% decline in surgical care during April of 2020 is likely owing to a combination of state-mandated cancellations of elective inpatient surgical procedures, more widespread stay-at-home social distancing orders, and self-imposed behavioral population-level mobility changes.^20^ Additionally, we found signs of a shift from inpatient to outpatient procedures during the pandemic, which warrants further research.

Both supply and demand factors for surgical care likely contributed to the declines in surgical use demonstrated by this study. The marked shortage of personal protective equipment, lack of early perioperative COVID-19 infection control guidelines, and state-mandated policies to create inpatient surge capacity for COVID-19 patients all contributed to decreased hospital supply of surgical care.^21,22^ From a demand perspective, the decrease in surgical use may be explained by patients’ confidence in health systems, as well as obstacles in scheduling surgeries. Prior literature has shown that the COVID-19 pandemic was associated with a decrease in outpatient in-person visits and an associated 23-fold increase in telehealth visits.^23,24^ Declines in outpatient primary care visits may have reduced referrals for surgical specialist consultation such as elective procedures for bariatric surgery or nonelective procedures for cancer. The effect of these broader outpatient trends on demand for surgical consultations and procedures warrants further investigation.

Overall, White patients had a greater decrease in surgical encounters across all surgical urgency cohorts. Given that encounters for traumatic illness and injury—which are mostly nondiscretionary—showed no statistically significant variation across racial and ethnic groups, the significant decrease in use of elective surgical procedures is suggestive of a discretionary nature of surgical use across racial and ethnic groups in the US during the COVID-19 pandemic. We had hypothesized that Black and Hispanic patients might have undergone a greater relative decline in use owing to unconscious bias in the prioritization of patients for constrained operating resources and exacerbations of underlying structural racism in access to care.^25,26,27^ However, findings of the opposite effect suggest a few potential mechanisms. First, there may be different preference sensitivity across racial and ethnic groups, as White patients have been associated with a greater degree of risk aversion in health care use.^28^ Therefore, surgical reductions during the pandemic may be more related to patient factors than surgical prioritization decisions. For acute care surgery, unconscious bias has not been shown to be a significant factor in treatment decisions, which may explain the small relative reductions in nonelective and urgent surgical encounters among racial and ethnic minority groups in this sample.^29^ Second, these findings could equally be owing to overuse of care among White patients at baseline before the pandemic. Across a variety of medical and surgical conditions, White patients have more use compared with minority patients.^30^ For elective procedures such as total joint arthroplasty, disparities in access to joint replacement may be mediated by preference sensitivity. For example, Black patients may be offered joint replacement at a more severe stage of disease and symptoms, and therefore, there may be less preference sensitivity on postponing surgery.^31^ Alternatively, given the location of surgical urgent care centers in more affluent areas, use of surgical procedures may be higher among White patients owing to more discretionary access due to better insurance coverage.^32^ Whether these patterns are owing to overuse of low-value elective surgical procedures among White patients or owing to reduced access to care among minority patients warrants further study, but the shock of the COVID-19 pandemic presents an unprecedented opportunity to understand the appropriateness and equity of the status quo of surgical care delivery prior to the COVID-19 pandemic.

### Limitations

There are various limitations of this study. First, PHD is an administrative database and relies on hospital reporting for accurate identification of surgical procedures, comorbidities, and demographics. While the PHD is broadly representative of US acute care hospitals, the patterns of surgical use may not apply to individual health systems. Second, PHD race and ethnicity information is self-reported, and approximately 10% of race and ethnicity information in this sample was other or unknown, which could indicate underrepresentation of racial and ethnic minority groups in the sample. Third, we could not determine causality in this observational study. Future studies will be needed to explore potential mechanisms for racial and ethnic variations in demand and preference sensitivity for elective surgical care during pandemics.

## Conclusions

In this cohort study of surgical encounters using a large, nationally representative hospital discharge-level data set, there was an approximately 13% decrease in overall surgical encounters during the COVID-19 pandemic and that surgical use did not fully recover to match baseline surgical use. White patients experienced the greatest relative reduction in utilization of elective, nonelective, and emergency surgical procedures during the pandemic, suggesting the possibility of greater preference sensitivity, risk aversion, or high baseline use among White patients compared with racial and ethnic minority patients. The lack of variation across racial and ethnic minority groups in encounters for trauma suggest that health systems were still able to provide equitable access to emergency department care. While COVID-19 had a disproportionate impact on underserved communities, our findings suggest that US health systems were still able to provide equitable access to surgical care during the COVID-19 pandemic.

## Notes

Supplement. eTable 1: Characteristics of Hospitals in the Premier Healthcare Database Compared to the American Hospital Association Annual Survey, 2018 eTable 2: Procedural codes of surgical cohorts eTable 3: Regression model estimates of change in surgical encounters by race and ethnicity and surgical urgency cohort, 2020 vs 2019 eFigure 1: Unadjusted total surgical encounters, 2020 vs 2019 eFigure 2: Change in surgical encounters by geographic region, 2020 vs 2019 eFigure 3: Change in surgical encounters by inpatient or outpatient status and surgical urgency cohort, 2020 vs 2019 eFigure 4: Change in surgical encounters by all races and ethnicities and surgical urgency cohort, 2020 vs 2019 eFigure 5: Change in surgical encounters by all races and ethnicities and surgical urgency cohort, 2020 vs 2019, with adjustments for hospital characteristics, COVID-19 case burden, and region
