# Supplementary material for: Variation in Use of Surgical Care During the COVID-19 Pandemic by Surgical Urgency and Race and Ethnicity
Source: JAMA Health Forum. 2021 Dec 23;2(12):e214214. doi: 10.1001/jamahealthforum.2021.4214 (PMC8796934; doi:10.1001/jamahealthforum.2021.4214)
Supplement: Supplement. — eTable 1: Characteristics of Hospitals in the Premier Healthcare Database Compared to the American Hospital Association Annual Survey, 2018 eTable 2: Procedural codes of surgical cohorts eTable 3: Regression model estimates of change in surgical encounters by race and ethnicity and surgical urgency cohort, 2020 vs 2019 eFigure 1: Unadjusted total surgical encounters, 2020 vs 2019 eFigure 2: Change in surgical encounters by geographic region, 2020 vs 2019 eFigure 3: Change in surgical encounters by inpatient or outpatient status and surgical urgency cohort, 2020 vs 2019 eFigure 4: Change in surgical encounters by all races and ethnicities and surgical urgency cohort, 2020 vs 2019 eFigure 5: Change in surgical encounters by all races and ethnicities and surgical urgency cohort, 2020 vs 2019, with adjustments for hospital characteristics, COVID-19 case burden, and region [file jamahealthforum-e214214-s001.pdf]

Supplemental Online Content

Tsai TC, Bryan AF, Rosenthal N, et al. Variation in use of surgical care during the COVID-19 pandemic by surgical urgency and race and ethnicity. *JAMA Health Forum*. 2021;2(12):e214214. doi:10.1001/jamahealthforum.2021.4214

eTable 1: Characteristics of Hospitals in the Premier Healthcare Database Compared to the American Hospital Association Annual Survey, 2018

eTable 2: Procedural codes of surgical cohorts
eTable 3: Regression model estimates of change in surgical encounters by race and ethnicity and surgical urgency cohort, 2020 vs 2019
eFigure 1: Unadjusted total surgical encounters, 2020 vs 2019
eFigure 2: Change in surgical encounters by geographic region, 2020 vs 2019
eFigure 3: Change in surgical encounters by inpatient or outpatient status and surgical urgency cohort, 2020 vs 2019
eFigure 4: Change in surgical encounters by all races and ethnicities and surgical urgency cohort, 2020 vs 2019
eFigure 5: Change in surgical encounters by all races and ethnicities and surgical urgency cohort, 2020 vs 2019, with adjustments for hospital characteristics, COVID-19 case burden, and region

This supplemental material has been provided by the authors to give readers additional information about their work.

© 2021 Tsai et al. *JAMA Health Forum.*

eTable 1: Characteristics of Hospitals in the Premier Healthcare Database Compared to the American Hospital Association Annual Survey, 2018

	PHD (2018)	AHA DATABASE (2018)*
	N (%)	N (%)
All Facilities	766 (100)	4,364(100)
**Provider Region**		
Midwest	204 (26.6)	1,320 (30.2)
Northeast	115 (15.0)	534 (12.2)
South	336 (43.9)	1,632 (37.4)
West	111 (14.5	878 (20.1)
**Rural/Urban**		
Rural	228 (29.8)	1,051 (24.1)
Urban	538 (70.2)	3,313(75.9)
**Beds**		
000-099	229 (29.9)	2,220 (50.9)
100-199	173 (22.6)	891 (20.4)
200-299	119 (15.5)	509 (11.7)
300-399	95 (12.4)	294 (6.7)
400-499	51 (6.7)	164 (3.8)
500+	99 (12.9)	286 (6.6)
**Teaching Status**		
Non-Teaching	549 (71.7)	2,582 (59.2)
Teaching	217 (28.3)	1,782 (40.8)

**Latest available data from the AHA*

© 2021 Tsai et al. *JAMA Health Forum.*

eTable 2: Procedural codes of surgical cohorts

	Procedure name	ICD-10 PCS codes	CPT codes	ICD-10 CM inclusion codes	ICD-10 CM exclusion codes
**Elective**					
	Sleeve gastrectomy	0DB64Z3	43775		C15*, C16*, C17*
	Gastric bypass	0D160ZB, 0D160ZA, 0D164ZB, 0D164ZA	43846, 43644		C15*, C16*, C17*
	Total hip arthroplasty	0SR90*, 0SRB0*	27130		S72.019A, S72.023A, S72.033A, S72.043A, S72.099A, S72.109A, S72.143A, S72.23XA, M84.459A
	Total knee arthroplasty	0SRD0*, 0SRC0*	27447		S72.90XA, S72.309A, S72.409A, S72.413A, S72.416A, S72.443A, S72.446A, S72.453A, S72.456A, S72.499A, M84.453A
	Ventral hernia repair	0WQF*, 0WUF*, 0KNK*, 0KNL*	15734, 49560, 49565, 49652, 49654, 49656	K43.2, K43.5, K43.9	K43.0, K43.1, K43.3, K43.4, K43.6, K43.7
	Myomectomy	0U590ZZ, 0U593ZZ, 0U594ZZ, 0U597ZZ, 0U598ZZ, 0UB90ZZ, 0UB93ZZ, 0UB94ZZ, 0UB97ZZ, 0UB98ZZ, 0U540ZZ, 0U543ZZ, 0U544ZZ, 0U548ZZ, 0UB40ZZ, 0UB43ZZ, 0UB44ZZ, 0UB47ZZ, 0UB48ZZ	58140, 58145, 58146, 58545, 58546	D25*, N84*, N92*, N93*	C53*, C54*, C55*, C56*, C57*, C58*

© 2021 Tsai et al. *JAMA Health Forum.*

	Hysterectomy	0UT90ZL, 0UT90ZZ, 0UTC0ZZ, 0UTC8ZZ, 0UT94ZL, 0UT94ZZ, 0UT97ZL, 0UT97ZZ, 0UT98ZL, 0UT98ZZ, 0UT9FZL, 0UT9FZZ	58541, 58542, 58543, 58544, 58570, 58571, 58572, 58573, 58260, 58262, 58263, 58267, 58270, 58290, 58291, 58292, 59293, 58294, 58570, 58571, 58572, 58573, 58550, 58551, 58552, 58553, 58554, 58541, 58542, 58543, 58544		C53*, C54*, C55*, C56*, C57*, C58*
	Breast reconstruction	0H0T07Z, 0H0T0JZ, 0H0T0KZ, 0H0T0ZZ, 0H0U07Z, 0H0U0JZ, 0H0U0KZ, 0H0U0ZZ, 0H0V07Z, 0H0V0JZ, 0H0V0KZ, 0H0V0ZZ, 0HHT0NZ, 0HHU0NZ, 0HHV0NZ, 0HPT0NZ, 0HPT3NZ, 0HPT7NZ, 0HPT8NZ, 0HPU0NZ, 0HPU3NZ, 0HPU7NZ, 0HPU8NZ, 0HRT075, 0HRU075, 0HRV075, 0HRT076, 0HRU076, 0HRV076, 0HRT077, 0HRU077, 0HRV077, 0HRT078, 0HRU078, 0HRV078, 0HRT079, 0HRU079, 0HRV079, 0HRT07Z, 0HRU07Z, 0HRV07Z, 0HRT0JZ, 0HRU0JZ, 0HRV0JZ, 0HRT0KZ, 0HRU0KZ, 0HRV0KZ, 0HST0ZZ, 0HSU0ZZ, 0HSV0ZZ, 0HUT07Z, 0HUT0JZ, 0HUT0KZ, 0HUU07Z, 0HUU0JZ, 0HUU0KZ, 0HUV07Z, 0HUV0JZ, 0HUV0KZ, 0J060ZZ, 0J063ZZ, 0JD60ZZ, 0JD63ZZ, 0KXK0Z6, 0KXL0Z6	19316, 19318, 19324, 19325, 19340, 19342, 19357, 19361, 19364, 19367, 19368, 19369, 19380	C50*, C79.81, D05*, N65*, Z42.1, Z85.3, Z90.1*	T81.4*, T85.4*, T85.79*, Z90.1*, S21.00*, S21.0*, S21.02*, S28.2*
	Ostomy closure	0DBB*, 0DBE*, 0DBF*, 0DBG*, 0DBK*, 0DBL*, 0DBM*, 0DBN*, 0DMB*, 0DME*, 0DMF*, 0DMG*, 0DMK*, 0DML*, 0DMM*, 0DMN*, 0WQF0ZZ	44227, 44620, 44625, 44626	Z43.3, Z93.2, Z93.3, Z43.2	
**Non-elective**					For all non-elective: exclude admissions via ED
	Mastectomy	0HBT*, 0HBU*, 0HBV*, 0HBW*, 0HBX*, 0HPT*, 0HPU*, 0HTT*, 0HTU*, 0HTV*, 0HTW*, 0HTX*	19301, 19302, 19303, 19304, 19305, 19306, 19307	C50*, C79.81, D05*, Z85.3	
	Radical prostatectomy	0VT00ZZ, 0VT04ZZ, 0VT07ZZ, 0VT08ZZ	55831, 55840, 55842, 55845, 55866	C61*, C77.5, C79.81, D07.5, D40.0, D49.5	

© 2021 Tsai et al. *JAMA Health Forum.*

	Pulmonary lobectomy, wedge/segmentectomy	0BTC0ZZ, 0BTC4ZZ, 0BTD0ZZ, 0BTD4ZZ, 0BTF0ZZ, 0BTF4ZZ, 0BTG0ZZ, 0BTG4ZZ, 0BTH0ZZ, 0BTH4ZZ, 0BTJ0ZZ, 0BTJ4ZZ, 0BBC0ZX, 0BBC0ZZ, 0BBC4ZX, 0BBC4ZZ, 0BBD0ZX, 0BBD0ZZ, 0BBD4ZX, 0BBD4ZZ, 0BBF0ZX, 0BBF0ZZ, 0BBF4ZX, 0BBF4ZZ, 0BBG0ZX, 0BBG0ZZ, 0BBG4ZX, 0BBG4ZZ, 0BBH0ZX, 0BBH0ZZ, 0BBH4ZX, 0BBH4ZZ, 0BBJ0ZX, 0BBJ0ZZ, 0BBJ4ZX, 0BBJ4ZZ, 0BBK0ZX, 0BBK0ZZ, 0BBK4ZX, 0BBK4ZZ, 0BBL0ZX, 0BBL0ZZ, 0BBL4ZX, 0BBL4ZZ, 0BBM0ZX, 0BBM0ZZ, 0BBM4ZX, 0BBM4ZZ	32505, 32506, 32507, 32607, 32608, 32666, 32667, 32668, 32480, 32482, 32484	C34*	
	Colectomy	0DTH4ZZ, 0DTF4ZZ, 0DTL4ZZ, 0DTG4ZZ, 0DTN4ZZ, 0DBE4ZZ, 0DBF4ZZ, 0DBG4ZZ, 0DBH4ZZ,0DBK4ZZ, 0DBL4ZZ, 0DBM4ZZ, 0DBN4ZZ, 0DTK4ZZ, 0DTM4ZZ, 0DBE0ZZ, 0DBE3ZZ, 0DBE7ZZ, 0DTH0ZZ, 0DTH7ZZ, 0DTF0ZZ, 0DTF7ZZ, 0DTK0ZZ, 0DTL0ZZ, 0DTL7ZZ, 0DTG0ZZ, 0DTG7ZZ, 0DTN0ZZ, 0DTN7ZZ, 0DBE0ZZ, 0DBE3ZZ, 0DBE7ZZ, 0DBF0ZZ, 0DBF3ZZ, 0DBF7ZZ, 0DBG0ZZ, 0DBG3ZZ, 0DBG7ZZ, 0DBH0ZZ, 0DBH3ZZ, 0DBH7ZZ, 0DBK0ZZ, 0DBK3ZZ, 0DBK7ZZ, 0DBL0ZZ<br>0DBL3ZZ, 0DBL7ZZ, 0DBM0ZZ, 0DBM3ZZ, 0DBM7ZZ, 0DBN0ZZ, 0DBN3ZZ, 0DBN7ZZ, 0DTK0ZZ, 0DTK7ZZ, 0DTM0ZZ, 0DTM7ZZ, 0DTE4ZZ, 0DTE0ZZ, 0DTE7ZZ	44140, 44141, 44143, 44144, 44145, 44146, 44147, 44150, 44151, 44155, 44156, 44157, 44160, 44204, 44205, 44206, 44207, 44208, 44210, 44211, 44212	C18*	K56*, A00*-A09*
	Aortic valve replacement	02RF37Z, 02RF38Z, 02RF3JZ, 02RF3KZ, 02RF07Z, 02RF08Z, 02RF0KZ, 02RF0JZ	33361, 33362, 33363, 33364, 33365, 33366, 33367, 33368, 33369, 33405, 33406, 33410, 33411, 33412, 33413	I06.0, I06.2, I08.8, I35.0, I35.1, I35.2, I35.8, I35.9, I42.1, Q23.0, Q24.4, Q25.3	I50.9
**Emergent**					
	Appendectomy	0DBJ*, 0DTJ*, 0D9J*, 0DQJ*, 0DTF*, 0DTH*, 0DTK*, 0D5J*, 0DCJ*, 0DDJ*	44950, 44955, 44960, 44870, 44979, 44900, 44901	K35*, K36*, K37*, K38*	C18*
	I&D of breast abscess	0H9T*, 0H9U*, 0H9V*, 0H9W*, 0H9X*	19020	N61.1	C50*, C79.81, D05*, Z85.3

© 2021 Tsai et al. *JAMA Health Forum.*

	Cholecystectomy	0F540ZZ, 0F543ZZ, 0FB40ZZ, 0FB43ZZ, 0FT40ZZ, 0FT44ZZ, 0F544ZZ, 0FB44ZZ	47562, 47563	K80.00, K80.01, K80.10, K80.11, K80.12, K80.13, K80.18, K80.19, K80.21, K80.80, K81.0, K81.1, K81.2, K81.9	C23*
	Colectomy for diverticulitis	0DTH4ZZ, 0DTF4ZZ, 0DTL4ZZ, 0DTG4ZZ, 0DTN4ZZ, 0DBE4ZZ, 0DBF4ZZ, 0DBG4ZZ, 0DBH4ZZ,0DBK4ZZ, 0DBL4ZZ, 0DBM4ZZ, 0DBN4ZZ, 0DTK4ZZ, 0DTM4ZZ, 0DBE0ZZ, 0DBE3ZZ, 0DBE7ZZ, 0DTH0ZZ, 0DTH7ZZ, 0DTF0ZZ, 0DTF7ZZ, 0DTK0ZZ, 0DTL0ZZ, 0DTL7ZZ, 0DTG0ZZ, 0DTG7ZZ, 0DTN0ZZ, 0DTN7ZZ, 0DBE0ZZ, 0DBE3ZZ, 0DBE7ZZ, 0DBF0ZZ, 0DBF3ZZ, 0DBF7ZZ, 0DBG0ZZ, 0DBG3ZZ, 0DBG7ZZ, 0DBH0ZZ, 0DBH3ZZ, 0DBH7ZZ, 0DBK0ZZ, 0DBK3ZZ, 0DBK7ZZ, 0DBL0ZZ<br>0DBL3ZZ, 0DBL7ZZ, 0DBM0ZZ, 0DBM3ZZ, 0DBM7ZZ, 0DBN0ZZ, 0DBN3ZZ, 0DBN7ZZ, 0DTK0ZZ, 0DTK7ZZ, 0DTM0ZZ, 0DTM7ZZ, 0DTE4ZZ, 0DTE0ZZ, 0DTE7ZZ	44140, 44141, 44143, 44144, 44145, 44146, 44147, 44150, 44151, 44155, 44156, 44157, 44160, 44204, 44205, 44206, 44207, 44208, 44210, 44211, 44212	K57*	C18*
	Incarcerated inguinal hernia repair	0YQ50ZZ, 0YQ5XZZ, 0YQ60ZZ, 0YQ6XZZ, 0YQAXZZ, 0YQA0ZZ, 0YQ53ZZ, 0YQ54ZZ, 0YQ63ZZ, 0YQ64ZZ, 0YQA4ZZ, 0YQA3ZZ	49507, 49521, 49650, 49651,	K40.0, K40.1, K40.3, K40.4	K40.2, K40.9
	Bowel resection for ischemia	0D8*, 0DB*, 0DC*, 0DD*, 0DF*, 0DM*, 0DN*, 0DP*, 0DQ*, 0DS*, 0DT*	44120, 44140	K55*	C18*
	Bowel resection for obstruction	0D8*, 0DB*, 0DC*, 0DD*, 0DF*, 0DM*, 0DN*, 0DP*, 0DQ*, 0DS*, 0DT*	44120, 44140	K56*	
**Trauma**		No procedure codes		S*, T07*, T14*, T2*, T30*, T31*, T32*, T79.A*	

© 2021 Tsai et al. *JAMA Health Forum.*

eTable 3: Regression model estimates of change in surgical encounters by race and ethnicity and surgical urgency cohort, 2020 vs 2019

Variable	Beta	Relative Risk	Relative Reduction (%)	SE	P-value
Intercept	6.251	518.635	-	0.105	<.0001
January	0.008	1.009	0.850	0.044	0.847
February	-0.052	0.949	-5.111	0.044	0.232
March	0.020	1.020	1.976	0.044	0.656
April	0.090	1.094	9.420	0.044	0.040
May	0.165	1.179	17.916	0.044	0.000
June	0.140	1.150	15.039	0.044	0.001
July	0.190	1.209	20.901	0.044	<.0001
August	0.176	1.193	19.256	0.044	<.0001
September	0.133	1.142	14.179	0.044	0.003
October	0.146	1.157	15.685	0.044	0.001
November	0.000	1.000	0.042	0.044	0.992
December	ref	ref	ref	ref	ref
Year	-0.211	0.809	-19.055	0.044	<.0001
Year*January	0.190	1.210	20.973	0.062	0.002
Year*February	0.181	1.198	19.806	0.062	0.004
Year*March	-0.044	0.957	-4.273	0.062	0.482
Year*April	-0.470	0.625	-37.500	0.062	<.0001
Year*May	-0.143	0.867	-13.299	0.062	0.022
Year*June	0.073	1.076	7.572	0.062	0.240
Year*July	0.058	1.060	5.963	0.062	0.351
Year*August	0.042	1.043	4.293	0.062	0.498
Year*September	0.061	1.063	6.298	0.062	0.325
Year*October	0.042	1.043	4.284	0.062	0.499
Year*November	0.044	1.045	4.515	0.062	0.476
Year*December	ref	ref	ref	ref	ref
White	ref	ref	ref	ref	ref
Black	-2.028	0.132	-86.838	0.045	<.0001
Hispanic	-2.353	0.095	-90.490	0.047	<.0001
Other	-2.625	0.072	-92.752	0.044	<.0001
Black*January	0.009	1.009	0.922	0.063	0.884
Black*February	0.001	1.001	0.087	0.063	0.989
Black*March	0.038	1.038	3.832	0.063	0.550
Black*April	0.008	1.009	0.851	0.063	0.893
Black*May	0.019	1.019	1.908	0.063	0.763
Black*June	0.013	1.014	1.352	0.063	0.831
Black*July	0.004	1.004	0.411	0.063	0.948
Black*August	-0.003	0.997	-0.258	0.063	0.967
Black*September	0.038	1.038	3.844	0.063	0.549
Black*October	0.034	1.034	3.423	0.063	0.592
Black*November	0.008	1.008	0.764	0.063	0.904
Black*December	0.000	1.000	0.000	ref	ref
Hispanic*January	-0.064	0.938	-6.168	0.065	0.330
Hispanic*February	-0.043	0.958	-4.203	0.065	0.512
Hispanic*March	0.016	1.016	1.577	0.065	0.811
Hispanic*April	0.015	1.015	1.496	0.065	0.820
Hispanic*May	0.023	1.023	2.321	0.065	0.725

© 2021 Tsai et al. *JAMA Health Forum.*

**Hispanic*June**	0.005	1.005	0.520	0.065	0.937
**Hispanic*July**	0.000	1.000	-0.035	0.065	0.996
**Hispanic*August**	0.019	1.019	1.871	0.065	0.776
**Hispanic*September**	0.031	1.031	3.099	0.065	0.640
**Hispanic*October**	0.056	1.058	5.785	0.065	0.389
**Hispanic*November**	0.025	1.025	2.519	0.065	0.703
**Hispanic*December**	ref	ref	ref	ref	ref
**Other*January**	-0.038	0.963	-3.704	0.062	0.545
**Other*February**	-0.052	0.949	-5.083	0.063	0.404
**Other*March**	-0.001	0.999	-0.070	0.062	0.991
**Other*April**	-0.003	0.997	-0.273	0.062	0.965
**Other*May**	-0.034	0.967	-3.340	0.062	0.585
**Other*June**	-0.031	0.969	-3.088	0.062	0.615
**Other*July**	-0.032	0.968	-3.175	0.062	0.605
**Other*August**	-0.027	0.973	-2.695	0.062	0.661
**Other*September**	-0.029	0.971	-2.896	0.062	0.637
**Other*October**	-0.009	0.991	-0.860	0.062	0.890
**Other*November**	-0.017	0.983	-1.693	0.062	0.784
**Other*December**	ref	ref	ref	ref	ref
**Year*White**	ref	ref	ref	ref	ref
**Year*Black**	0.005	1.005	0.477	0.063	0.940
**Year*Hispanic**	0.006	1.006	0.577	0.065	0.930
**Year*Other**	-0.030	0.971	-2.919	0.062	0.635
**Year*Black*January**	-0.017	0.983	-1.668	0.089	0.850
**Year*Black*February**	0.022	1.022	2.247	0.089	0.803
**Year*Black*March**	-0.020	0.980	-1.970	0.089	0.823
**Year*Black*April**	-0.105	0.900	-9.959	0.089	0.239
**Year*Black*May**	-0.131	0.878	-12.234	0.089	0.143
**Year*Black*June**	-0.082	0.921	-7.888	0.089	0.355
**Year*Black*July**	-0.068	0.935	-6.535	0.089	0.447
**Year*Black*August**	-0.034	0.966	-3.390	0.089	0.698
**Year*Black*September**	-0.057	0.944	-5.583	0.089	0.518
**Year*Black*October**	-0.032	0.969	-3.134	0.089	0.720
**Year*Black*November**	0.015	1.015	1.463	0.089	0.870
**Year*Black*December**	ref	ref	ref	ref	ref
**Year*Hispanic*January**	0.086	1.090	9.016	0.092	0.349
**Year*Hispanic*February**	0.112	1.119	11.885	0.092	0.224
**Year*Hispanic*March**	0.029	1.030	2.955	0.092	0.752
**Year*Hispanic*April**	-0.042	0.959	-4.088	0.092	0.651
**Year*Hispanic*May**	-0.099	0.906	-9.389	0.092	0.284
**Year*Hispanic*June**	-0.081	0.922	-7.816	0.092	0.377
**Year*Hispanic*July**	-0.098	0.907	-9.333	0.092	0.287
**Year*Hispanic*August**	-0.063	0.939	-6.073	0.092	0.496
**Year*Hispanic*September**	-0.046	0.955	-4.476	0.092	0.618
**Year*Hispanic*October**	-0.077	0.926	-7.375	0.092	0.404
**Year*Hispanic*November**	-0.003	0.997	-0.312	0.092	0.973
**Year*Hispanic*December**	ref	ref	ref	ref	ref
**Year*Other*January**	0.025	1.025	2.510	0.088	0.779
**Year*Other*February**	0.077	1.080	7.950	0.088	0.387
**Year*Other*March**	-0.015	0.985	-1.508	0.088	0.863

© 2021 Tsai et al. *JAMA Health Forum.*

**Year*Other*April**	-0.055	0.947	-5.349	0.088	0.534
**Year*Other*May**	-0.045	0.956	-4.419	0.088	0.608
**Year*Other*June**	-0.034	0.967	-3.350	0.088	0.699
**Year*Other*July**	-0.001	0.999	-0.070	0.088	0.994
**Year*Other*August**	0.013	1.013	1.323	0.088	0.882
**Year*Other*September**	0.008	1.009	0.850	0.088	0.923
**Year*Other*October**	-0.019	0.981	-1.864	0.088	0.831
**Year*Other*November**	0.024	1.024	2.386	0.088	0.789
**Year*Other*December**	ref	ref	ref	ref	ref

Model estimates from a multi-variable generalized linear model with a gamma distribution and log link with hospital fixed effects. The analyses compare rates of surgical encounters by hospital month in 2020 vs 2019. Coefficients for hospital fixed effects are not displayed for ease of presentation. To obtain the relative reduction metrics presented in Figure 3, model coefficients were summed, exponentiated and then converted into a year over year percent relative reduction.

© 2021 Tsai et al. *JAMA Health Forum.*

eFigure 1: Unadjusted total surgical encounters, 2019 vs 2020

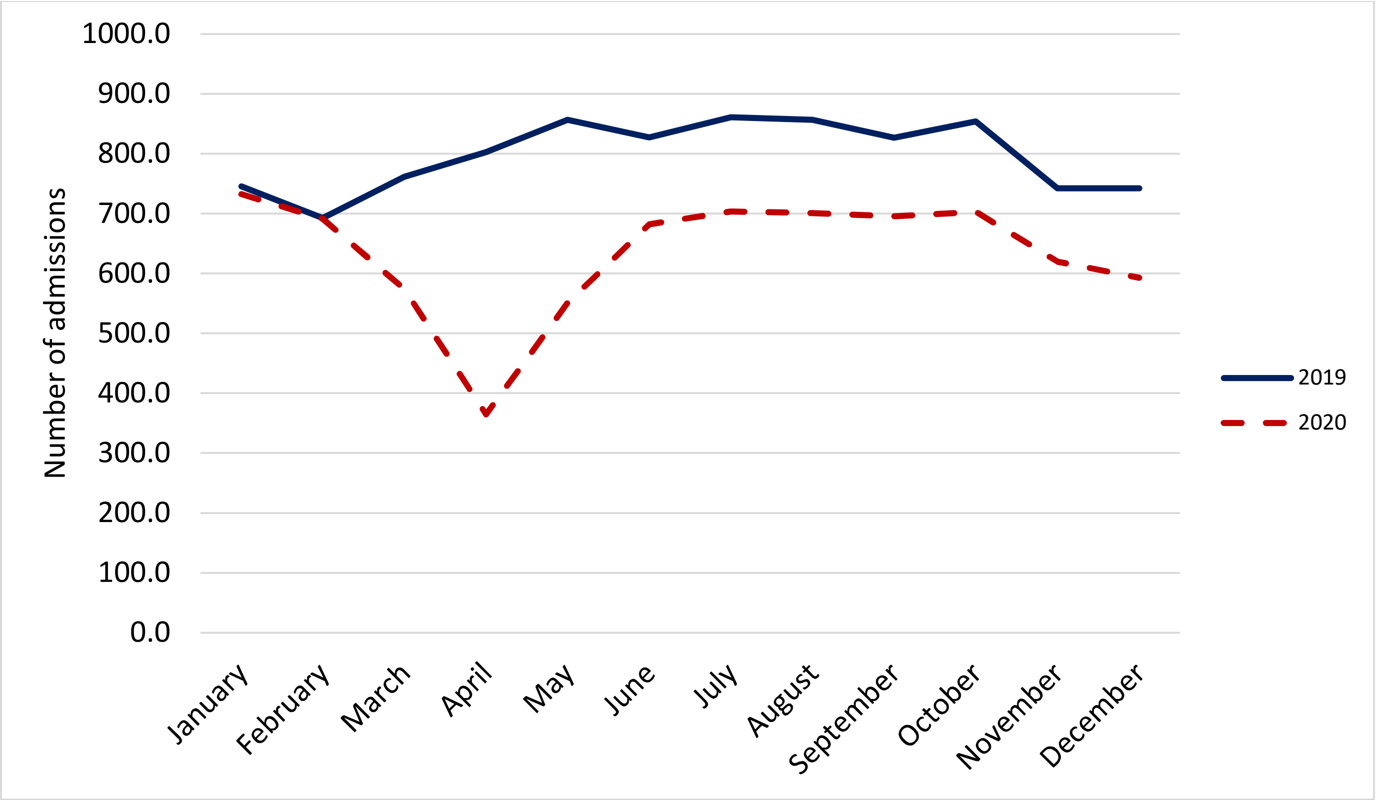

© 2021 Tsai et al. *JAMA Health Forum.*

eFigure 2: Change in surgical encounters by geographic region, 2019 vs 2020

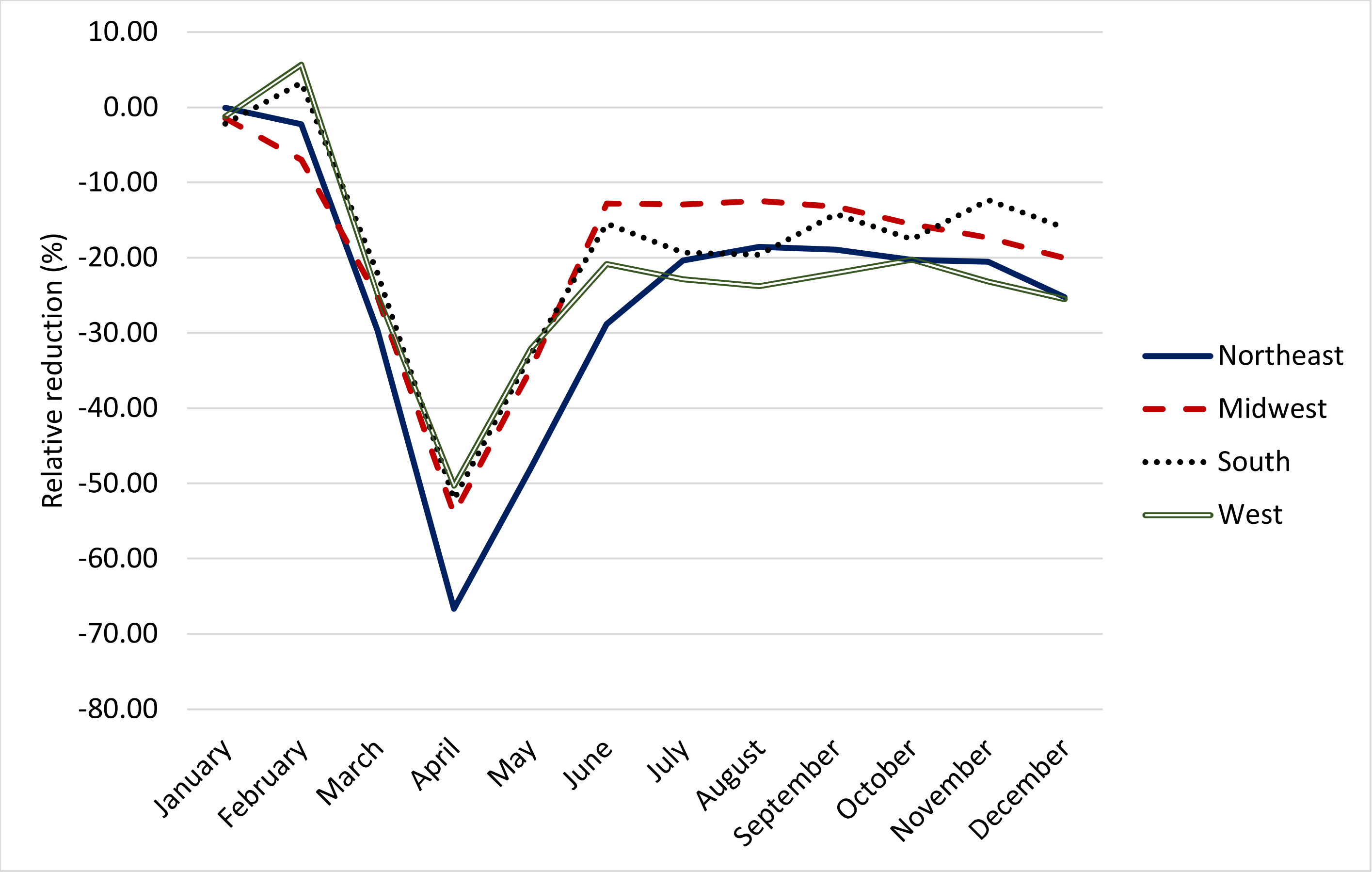

© 2021 Tsai et al. *JAMA Health Forum.*

eFigure 3: Change in surgical encounters by inpatient or outpatient status and surgical urgency cohort, 2019 vs 2020

3a. Inpatient Surgical Encounters

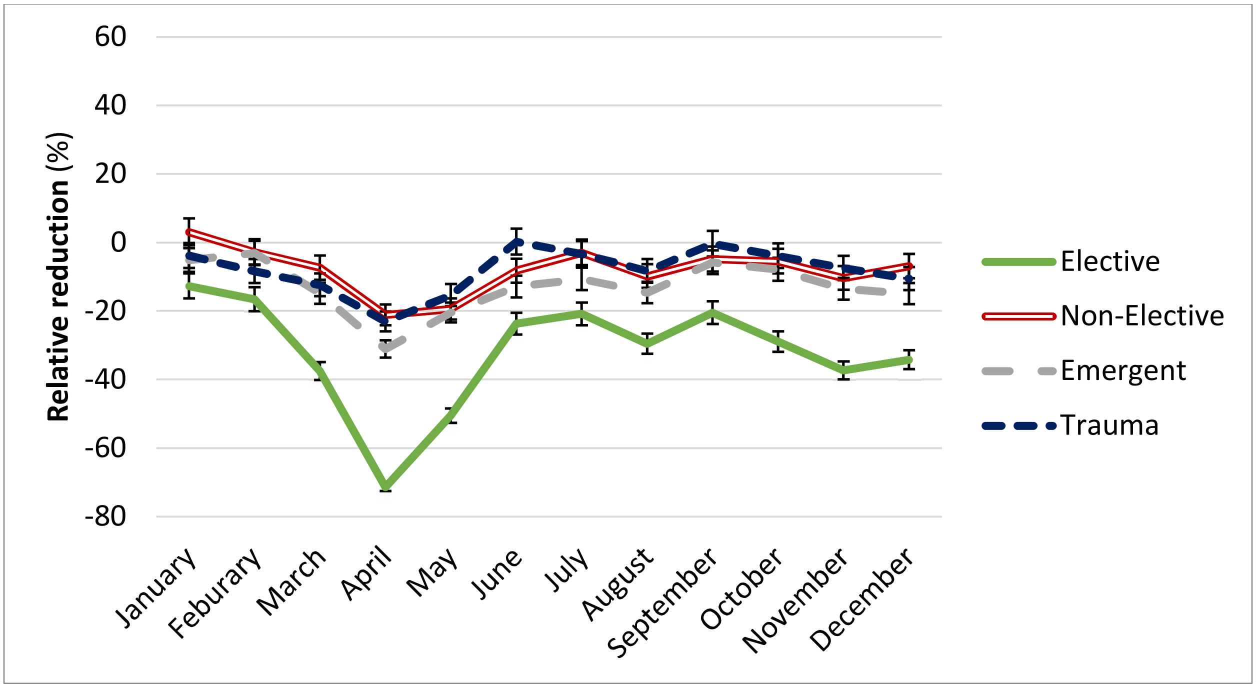

© 2021 Tsai et al. *JAMA Health Forum.*

3b. Outpatient Surgical Encounters

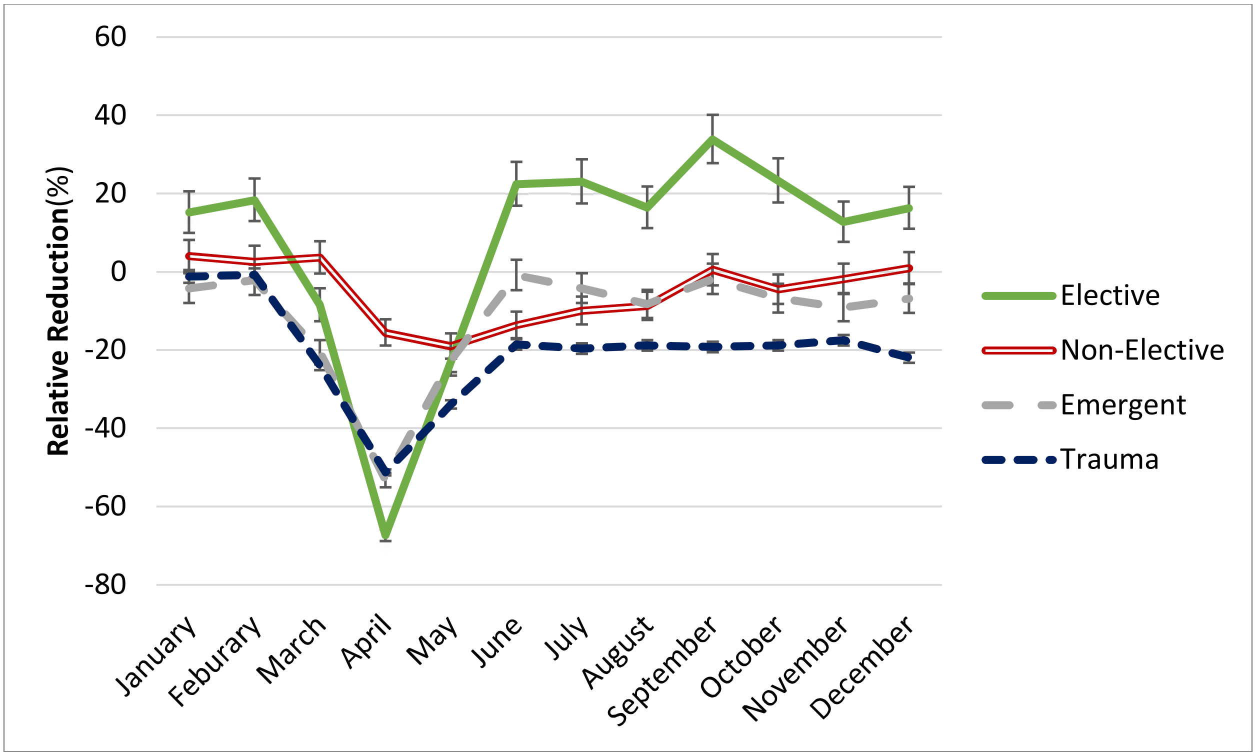

© 2021 Tsai et al. *JAMA Health Forum.*

eFigure 4: Change in surgical encounters by race and ethnicity and surgical urgency cohort, 2019 vs 2020

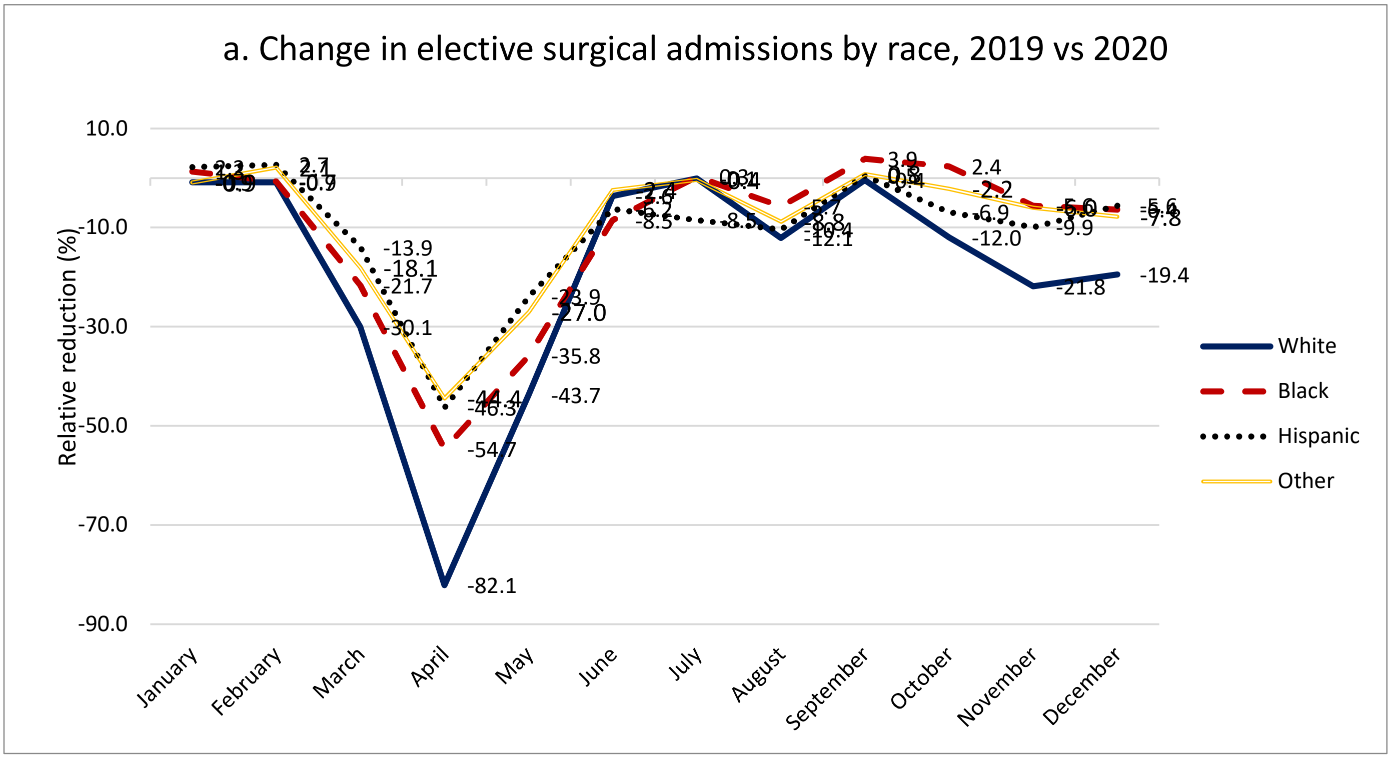

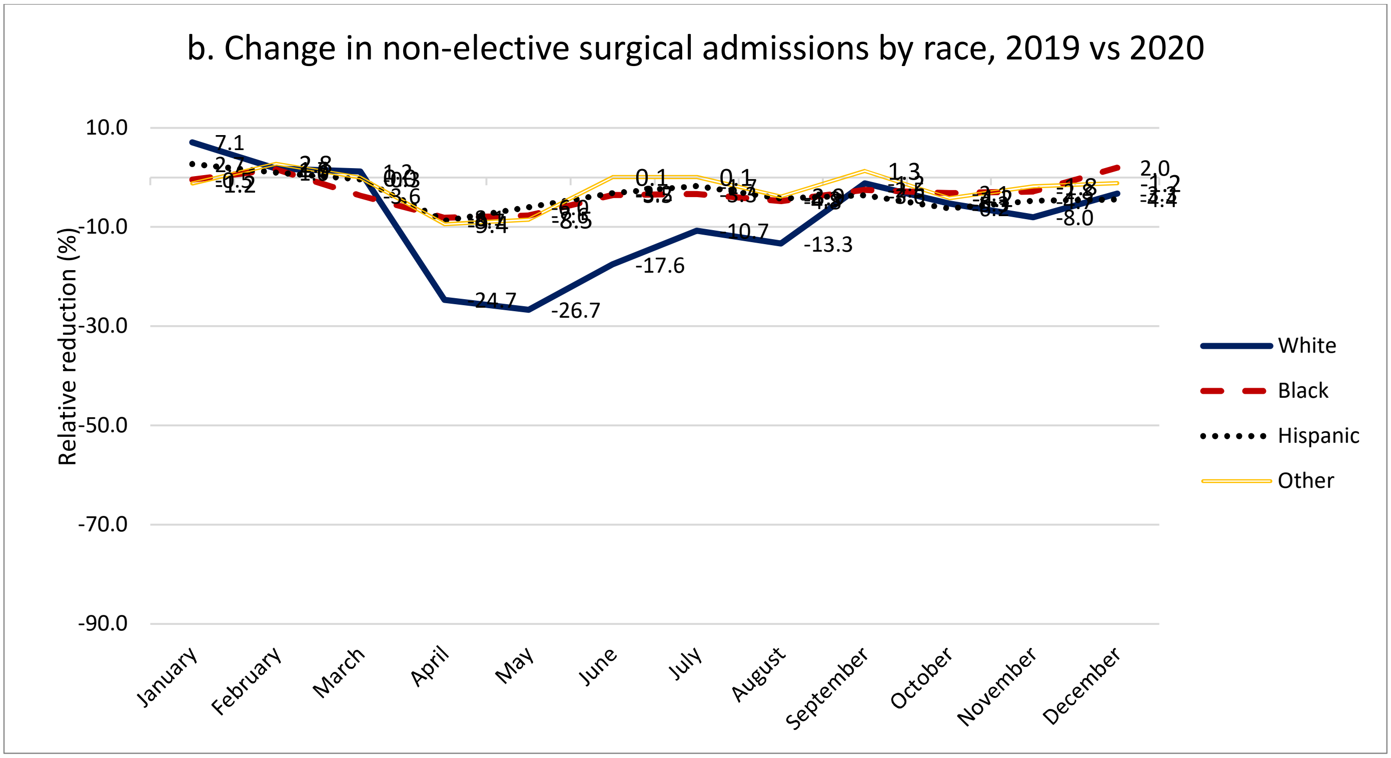

© 2021 Tsai et al. *JAMA Health Forum.*

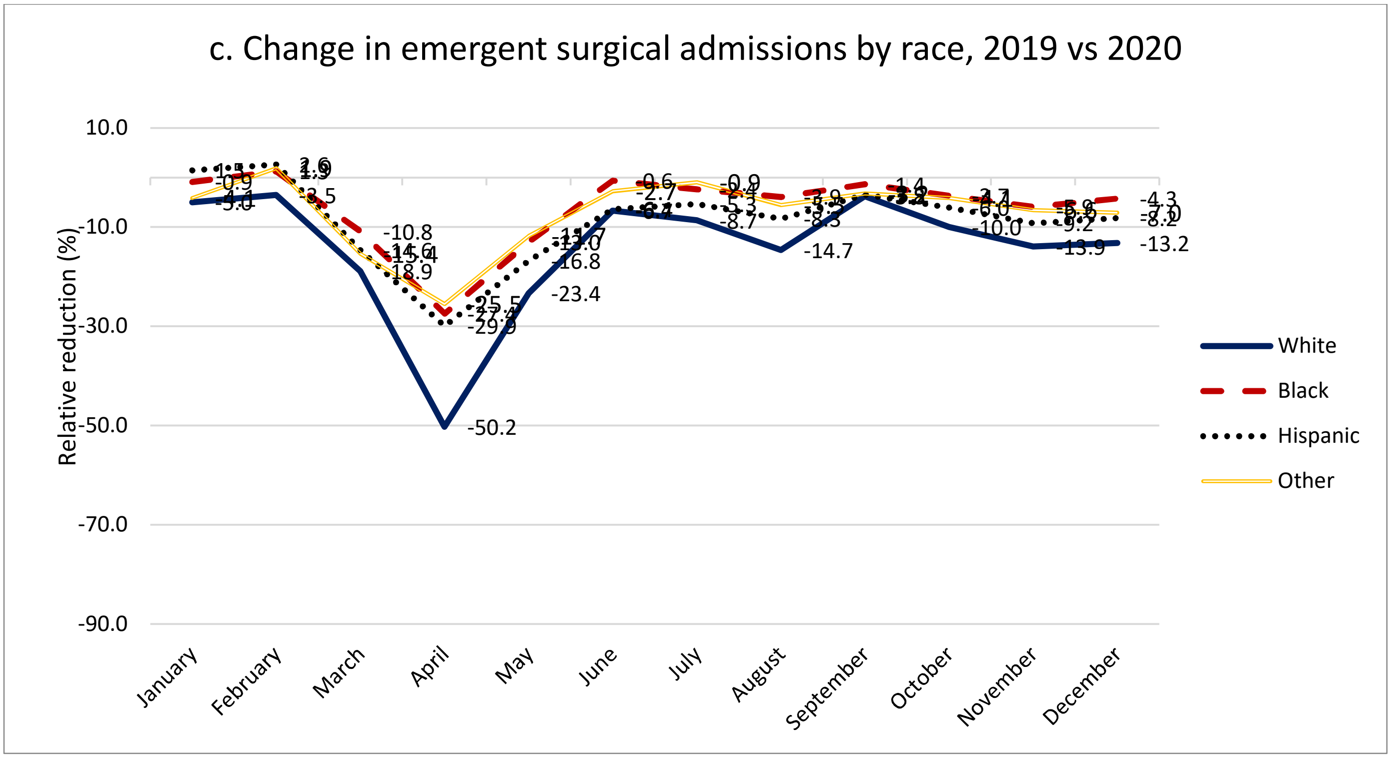

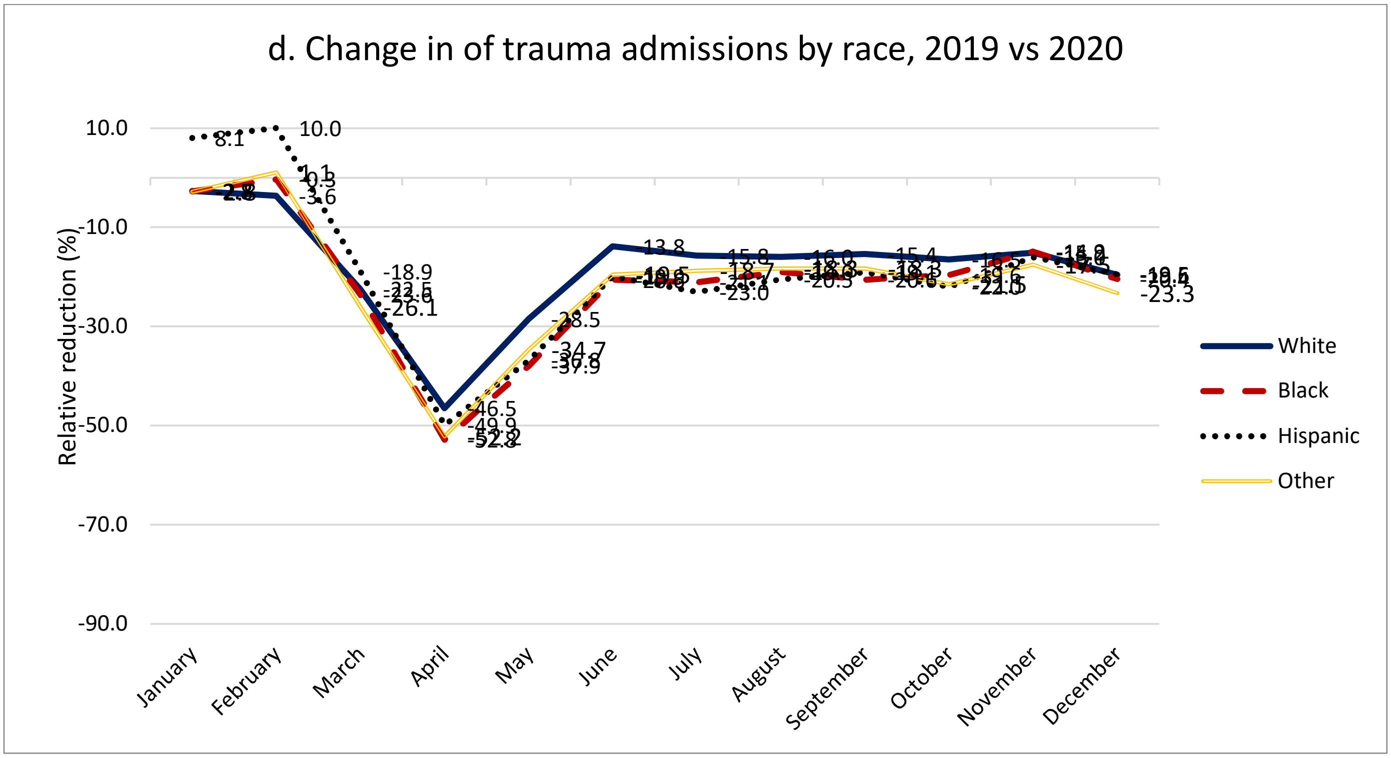

© 2021 Tsai et al. *JAMA Health Forum.*

eFigure 5: Change in surgical encounters by race and ethnicity and surgical urgency cohort, 2019 vs 2020, with adjustments for hospital characteristics, COVID-19 case burden, and region

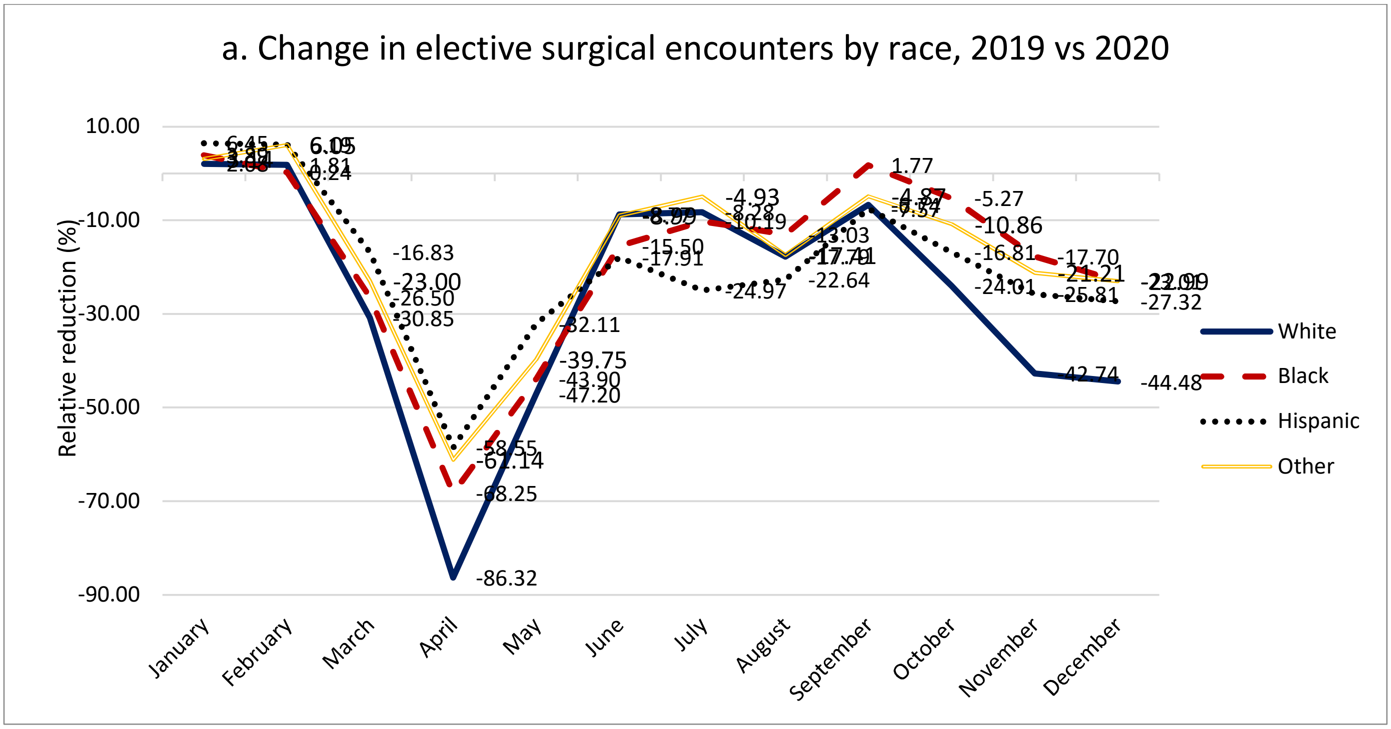

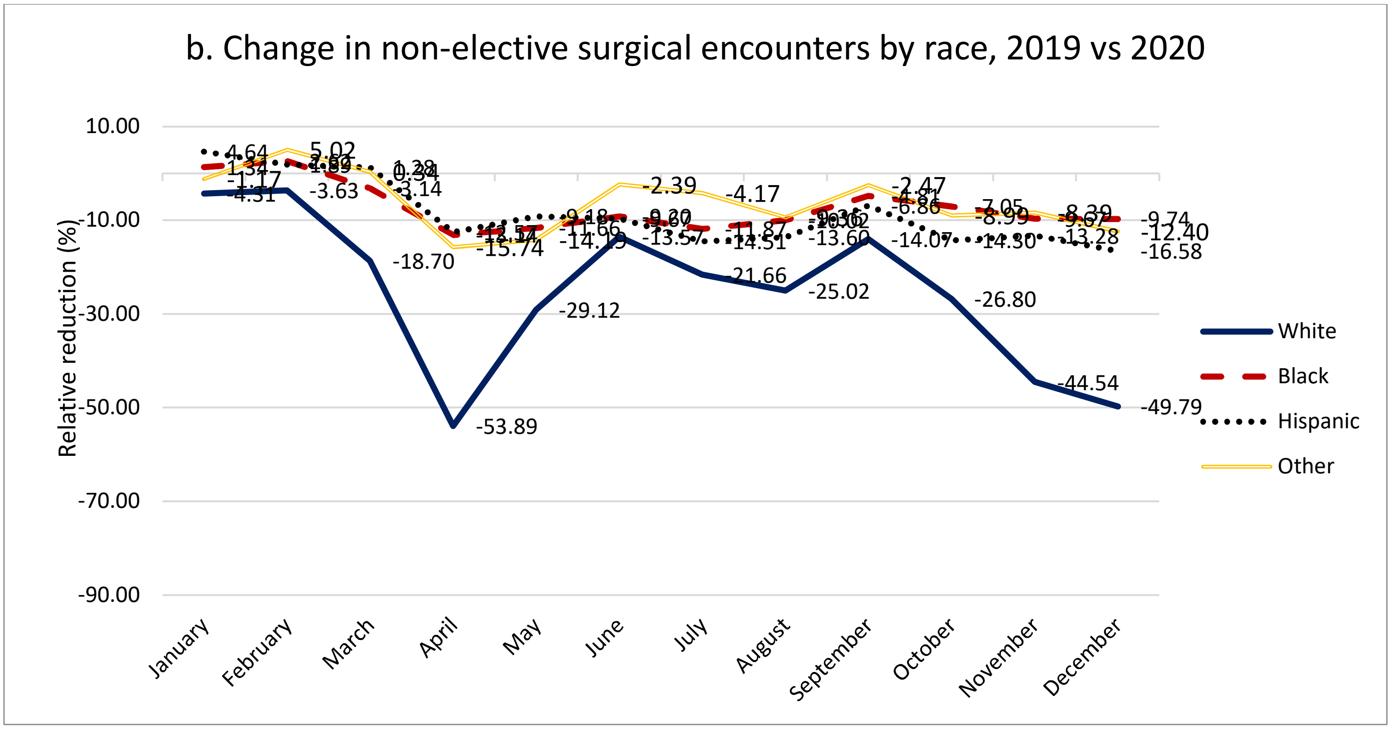

© 2021 Tsai et al. *JAMA Health Forum.*

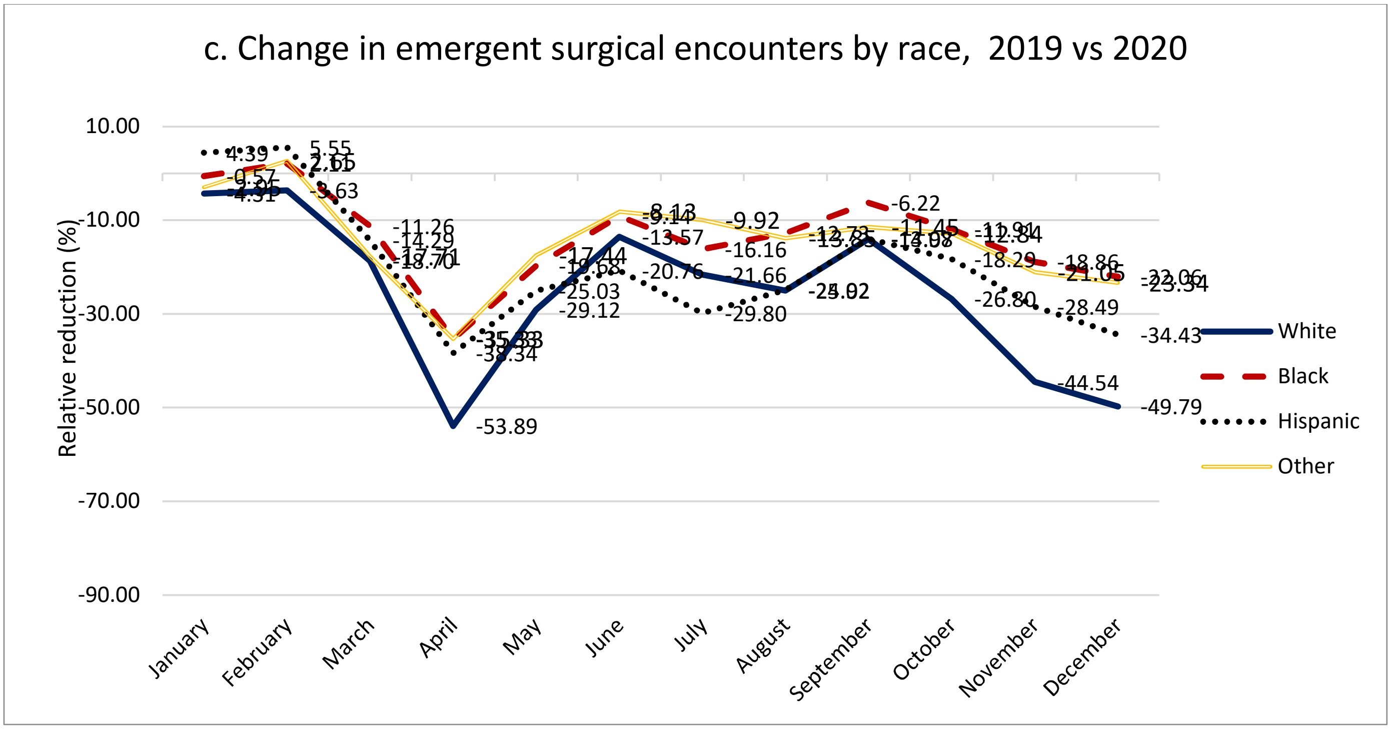

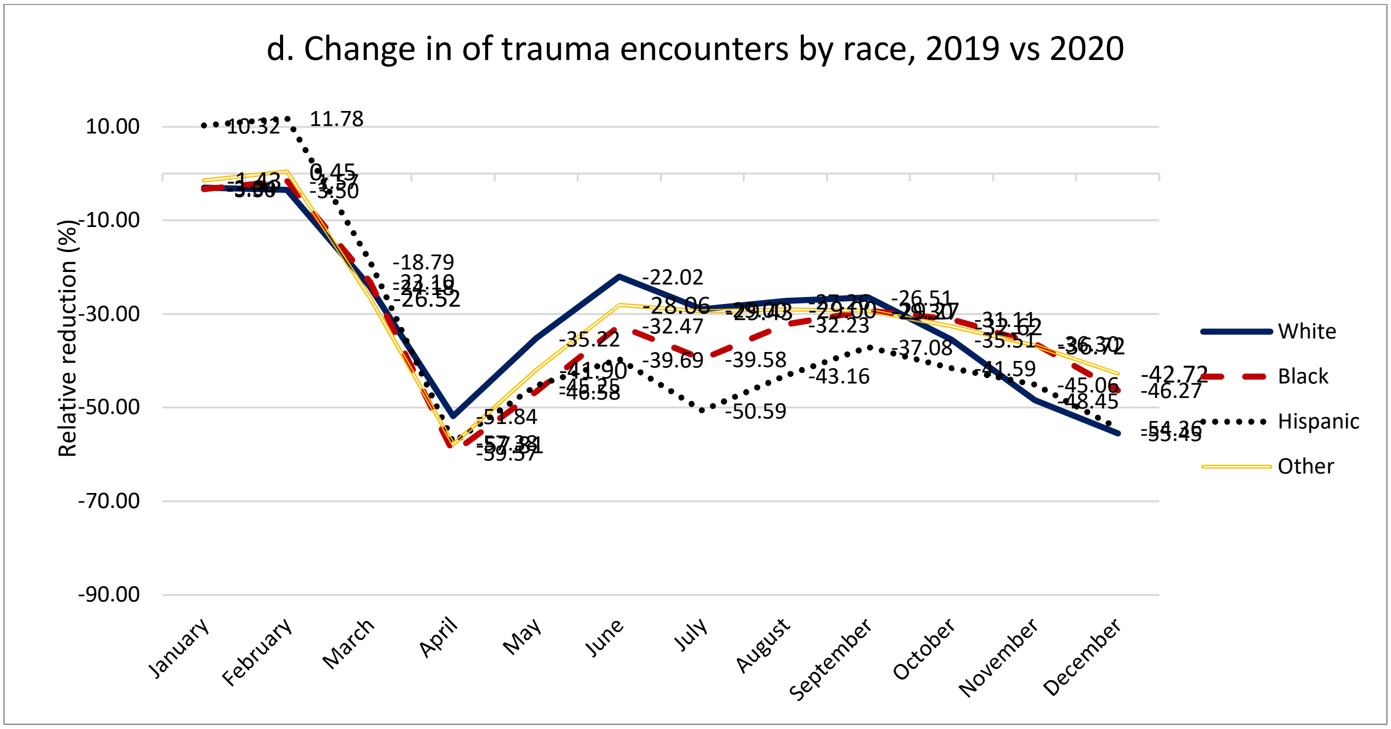

© 2021 Tsai et al. *JAMA Health Forum.*
